# Abscess of the Larynx

**Published:** 1829-06

**Authors:** 


### HOSPITAL REPORTS,

(*Principally condensed from various Periodical Publications.*)

---

### ABSCESS OF THE LARYNX.

*Abscess of the Larynx. Sudden and unexpected Death. Dissection.* (Hôpital de la Charité.)

A robust man, a cartwright by trade, æt. twenty-eight, had felt indisposed for some days. He had lost his appetite, and had had pains in his limbs. Admitted April 8th, 1829. His face was of a yellowish cast, particularly around the nose and lips. Dull countenance. Vomiting. Pulse quicker than natural.

M. Cayol could detect no local disease, and for two days the case was left to nature. As the patient now was much oppressed, two grains of emetic tartar were given. Bilious vomiting followed. Continued better until the 17th. At this time an attack of fever occurred, and the feelings of general uneasiness were increased. The patient had a very unfavorable appearance. Bleeding from the nose, but no alleviation of the symptoms. A troublesome cough soon came on, attended by difficult respiration and sanguineous expectoration. The stethoscope was applied, but only the mucous rattle was detected. The students had observed some attacks threatening suffocation.

18th.—An aphthous appearance on the left tonsil; redness of the palate; great depression and difficulty of swallowing, even the saliva.

19th.—Difficulty of breathing increased. M. Cayol, with the view of remedying pulmonary catarrh, ordered two ipecacuan emetics.

20th.—Had vomited. Bowels freely open. Appears very easy.

He rose up suddenly, placed his hand upon the sternum, and cried out that he should be suffocated. M. Miguel immediately attended, and found the patient in a state of asphyxia. He prepared to bleed him, but he died instantly. Five minutes before, he had conversed without difficulty with the nurse.

*Dissection, twenty-two hours post mortem.*—No appearances were detected in the brain, thoracic or abdominal viscera, which could be connected with the symptoms that had existed. The tongue was very thick, particularly towards its base. The mucous membrane of the pharynx and palate was very red; and the membrane covering the epiglottis, the ligaments, and the entrance of the larynx, were also red and thickened. This redness did not pass beyond the lips of the glottis. The edges of the epiglottis were swollen. There was an aphthous appearance on the left tonsil, and upon the epiglottis a membranous secretion, which closely adhered to the mucous membrane, and resembled that which is found upon the tonsils at the commencement of the disease which has been described by M. Bretonneau under the name of *diptherite*. But the most remarkable morbid appearance was the following: There was a partial destruction of, and an aperture in, the mucous membrane a little on the outside of the arytæno-epiglottic ligament. Purulent serum was discharged from the aperture, upon pressure. Upon enlarging this opening, a collection of matter was discovered around the base of the tongue. The subcutaneous cellular tissue which covers the epiglottis and glottis was thickened, and full of serum. It did not contain any pus.*

---

